# IL-6 Secreted from Senescent Mesenchymal Stem Cells Promotes Proliferation and Migration of Breast Cancer Cells

**DOI:** 10.1371/journal.pone.0113572

**Published:** 2014-11-24

**Authors:** Guo-hu Di, Yang Liu, Ying Lu, Jin Liu, Chutse Wu, Hai-Feng Duan

**Affiliations:** 1 Beijing Institute of Radiation Medicine (BIRM), No. 27, Taiping Road, Haidian District, Beijing 100850, China; 2 The 307 Hospital, No. 8, Dongdajie Street, Fengtai District, Beijing 100071, China; 3 State Key Laboratory Cultivation Base, Shandong Provincial Key Laboratory of Ophthalmology, Shandong Eye Institute, Shandong Academy of Medical Sciences, 5 Yan'erdao Road, Qingdao 266071, China; Baylor College of Medicine, United States of America

## Abstract

Human mesenchymal stem cells (hMSCs) are currently investigated for a variety of therapeutic applications. However, MSCs isolated from primary tissue cannot meet clinical grade needs and should be expanded in vitro for several passages. Although hMSCs show low possibility for undergoing oncogenic transformation, they do, similar to other somatic cells, undergo cellular senescence and their therapeutic potential is diminished when cultured in vitro. However, the role of senescent MSCs in tumor progression remains largely elusive. In the current study, by establishing senescent human umbilical cord mesenchymal stem cells (s-UCMSCs) through the replicative senescence model and genotoxic stress induced premature senescence model, we show that s-UCMSCs significantly stimulate proliferation and migration of breast cancer cells in vitro and tumor progression in a co-transplant xenograft mouse model compared with ‘young’ counterparts (defined as MSCs at passage 5, in contrast to senescent MSCs at passage 45). In addition, we identified IL-6, a known pleiotropic cytokine, as a principal mediator for the tumor-promoting activity of s-UCMSCs by induction of STAT3 phosphorylation. Depletion of IL-6 from s-UCMSCs conditioned medium partially abrogated the stimulatory effect of s-UCMSCs on the proliferation and migration of breast tumor cells.

## Introduction

Mesenchymal stromal cells (MSCs) represent heterogeneous subsets of a multipotent cell population that, given the appropriate condition, have the capability to differentiate into different mesoderm-derived cell lineages, including chondrocytes, osteocytes, and adipocytes [1]. Based on their ease of culture expansion, multilineage differentiation potential and potent immuno-regulatory properties, MSCs hold great promise for regenerative medicine and for treating severe immune-mediated disorders [2]–[4]. Currently, more than 300 clinical trials evaluating MSC therapy are registered on ClinicalTrials.gov and no critical side effects have yet been described.

As the low frequency of mesenchymal stem cells in the primary tissue, it always necessitates in vitro expansion before clinical use. Safety and functionality of cultured MSCs are main concerns for cellular therapy [5]–[7]. Although the standardized culture conditions or specific criteria has been released, it has been proven that donor validation, choice of isolation or culture conditions could have practical implications on the functional properties and therapeutic potential of clinical-grade MSCs [8], [9].

Similar to other adult somatic cells, MSCs could be subject to the irreversible arrest of cell division due to intrinsic factors or induction by the somatic environment within 2–3 months [10]. In fact, MSCs might enter senescence almost undetectably from the moment in vitro culture begins. Cells display a characteristic enlarged, flattened morphology, slowed proliferation rate, shortened telomere length and alteration of their secretory profile. Simultaneously, MSCs are losing their multipotential characteristics. It has been previously described early on that the differentiation potential to form an osteocyte declines during late-passages, which may be a cause of senile osteoporosis [11]. Other studies have demonstrated that the impairment on adipogenic potential may be even greater [12]. Moreover, mesenchymal stem cells from aged donors have reduced wound repair, angiogenesis and tissue homeostasis reconstruction capabilities [13].

As a critical component of the tissue microenvironment, MSCs have attracted great interest in breast cancer research because of their marked tropism for tumors and ability to integrate into tumor stroma [14]. Moreover, MSCs that reside in the stroma of breast cancer could enhance tumor metastases via the CCL5/CCR signaling pathway [15].

Senescent cells can alter the tissue microenvironment and affect nearby malignant epithelial cells [16]. However, whether senescent-related changes in MSCs could alter their interaction within a tumor or comparison between ‘young’ MSCs and senescent MSCs during mammary carcinoma progression remains inconclusive. In this study, we show that in comparison with ‘young’ MSCs, senescent human umbilical cord mesenchymal stem cells (s-UCMSCs) significantly promote the proliferation and migration of breast cancer cells through the IL-6/STAT-3-dependent pathway. Our data suggest that a cellular senescence evaluation for MSCs should be implemented before clinical application.

## Materials and Methods

### Cell culture and preparation of conditioned medium

UCMSCs were isolated, cultured and characterized as described before in accordance with the Ethics Committee at the Beijing Institute of Radiation [17]. Human breast cancer cell lines, MDA-MB-231 and MCF-7, were maintained in DMEM (Invitrogen, America) medium supplemented with 10% FBS and 1× Pen/Strep (Invitrogen). To induce replicative senescence, MSCs were passaged serially to over 40 passages when the typical senescence-associated features appeared. To induce oxidative stress, cells were incubated for 2 h in the presence of hydrogen peroxide (200 µM), and then washed and incubated in fresh medium [18]. Senescence-associated β-galactosidase (SA-β-Gal) staining was performed using an SA-β-Gal staining kit (Cell Signaling) according to the manufacturer's instructions.

To prepare the conditioned medium (CM), senescent and young MSCs (1×10^5^ per well) were plated in 6-well plates and incubated overnight. The medium was replaced with 2 ml per well of fresh serum-free DMEM and cultured for 24 h. The medium was then collected, centrifuged at 1000 rpm for 5 min, filtered through a 0.22 µm membrane and preserved at −80°C until use.

### Osteogenic, adipogenic and Chondrogenic differentiation

For osteogenic differentiation, MSCs were seeded into a 24-well culture plates at a density of 5000 cells per well and induced with DMEM containing 10% FBS, 0.1 µM dexamethasone (Sigma), 50 µM ascorbic acid (Sigma), and 10 mM β-glycerophosphate (Sigma). The medium was changed every 3 days. After 3 weeks, cells were fixed with 10% formalin for 20 min and stained with Alkaline phosphatase(ALP) (Sigma).

For adipogenic differentiation, MSCs were seeded in a 24-well plate at a density of 1×10^4^ cells per well and induced with DMEM containing 10% FBS, 1 µM dexamethasone, 5 µg/mL insulin (Sigma), and 100 µM indomethacin (Sigma). The medium was changed every 3 days. After 3 weeks, cells were fixed with 10% formalin for 20 min, and intracellular lipid droplets were revealed by Oil-Red (Sigma) staining.

Chondrogenic differentiation was seeded in a 24-well plate at a density of 1×10^4^ cells per well at 37°C for 14 days in chondrogenic medium consisting of DMEM-HG with 0.1 µM dexamethasone (Sigma), 1 mM sodium pyruvate, 170 µM ascorbic acid-2-phosphate (Sigma), 1× insulin-transferrin-selenium (Sigma), and 10 ng/mL transforminggrowth factor-beta-1 (TGF-b1) (AbCys). Medium was changed every 3 days. Chondrogenic differentiation was evaluated after 14 days of induction. The presence of glycosaminoglycans in cell pellets was revealed by Toluidine blue staining.

### Cell proliferation assay

Breast cancer cells (MDA-MB-231 and MCF-7) were seeded in 96-well plates at a density of 2×10^3^ per well and allowed to adhere for 12 h. Cells were then serum starved in serum-free DMEM for 24 h. Next, the medium was replaced with conditioned medium from MSCs and cultured for another 48 h. The proliferation of tumor cells was evaluated using a CCK-8 kit (Dojindo, Japan) and performed according to the manufacturer's instructions.

### Cell migration assay

To perform the migration assays, 24-well transwell chambers (8 µm pore; Corning, NY) were utilized. Mesenchymal stem cells (2×10^5^ per well) were loaded into the lower room for adherence and incubated overnight. Medium was then changed to DMEM containing 10% FBS and cultured for 24 h. Breast cancer cells (MDA-MB-231 and MCF-7; 5×10^4^ per well) were suspended in serum-free DMEM, added to the upper chamber and incubated for 12 h. Non-migrating cells were removed from the top chamber using a cotton swab. The cells remaining in the bottom chamber were fixed with 4% paraformaldehyde for 15 min and stained with 0.1% crystal violet in 2% ethanol. The cells that migrated through the porous membrane were visually quantified in 3–5 random fields from each membrane under a microscope. All experiments were performed at least three times.

### Enzyme-linked immunosorbent assay

MSCs (1×10^5^ per well) were plated in 6-well plates and 12 hours later, the sub-confluent cells were starved in serum-free DMEM. The medium was replaced with 2 ml per well of fresh serum-free DMEM, and the culture supernatants were collected after 24 h. IL-6, HGF, VEGF and TNF-α secretion in the supernatants were measured using ELISA according to the manufacturer's protocols (R&D Systems, America).

### Western blot

Breast cancer cells (MDA-MB-231 and MCF-7) were seeded into 6-well plates and starved for 24 h. Conditioned medium from senescent MSCs with or without the mouse monoclonal anti-hIL6-antibody was added and incubated for 30 mins. A mouse monoclonal IgG was used as control. Cells were harvested and lysed by RIPA buffer containing a cocktail of protease inhibitors and phosphatase inhibitors. After electrophoretic separation and immunoblotting of whole tumor cell lysates, p-STAT3 (Y705) was detected with a mouse monoclonal anti-p-STAT3 antibody. STAT3 or GAPDH were used as controls (Cell Signaling Technology, America).

### Animal model and immunohistochemical analysis

Female immunodeficient mice (Laboratory Animal Center, Academy of Military Medical Sciences, Beijing, China) at the age of 8 weeks with an average weight of approximately 20 g were used in this experiment. Procedures were carried out with approval from the Animal Use and Care Committee of Beijing Institute of Radiation Medicine (Permit Number: 2012005). Mice were diveded into three groups (n = 8 per group). Cell transplantations were performed as previously described [19]. Either MDA-MB-231 (2×10^6^ cells) alone or mixed with an equal number of H_2_O_2_-induced MSCs or passage 5 MSCs were subcutaneously injected in the right flank region of the animals. Tumors were measured every 7 days using a digital vernier caliper, and the tumor volume was calculated as (length×width^2^)/2. On day 42, mice were sacrificed by cervical dislocation humanely. Subcutaneous tumors werev removed and weighed before they were fixed in 4% paraformaldehyde for histological examination. The immunohistochemical experiment for endothelin-1 (1∶100, Abcam) was executed as previously described [19]. Blood vessels were counted under 100× magnification.

### Statistical analysis

The data are represented as the mean ± standard deviation (SD). Comparisons between two groups were performed using Student's t-test, and one-way ANOVA was used for multiple comparisons. A *P*-value<0.05 was considered to be significant.

## Results

### Confirmation of UCMSC senescence using replicative exhaust or a sub-lethal dose of H_2_O_2_

**Figure 1:**
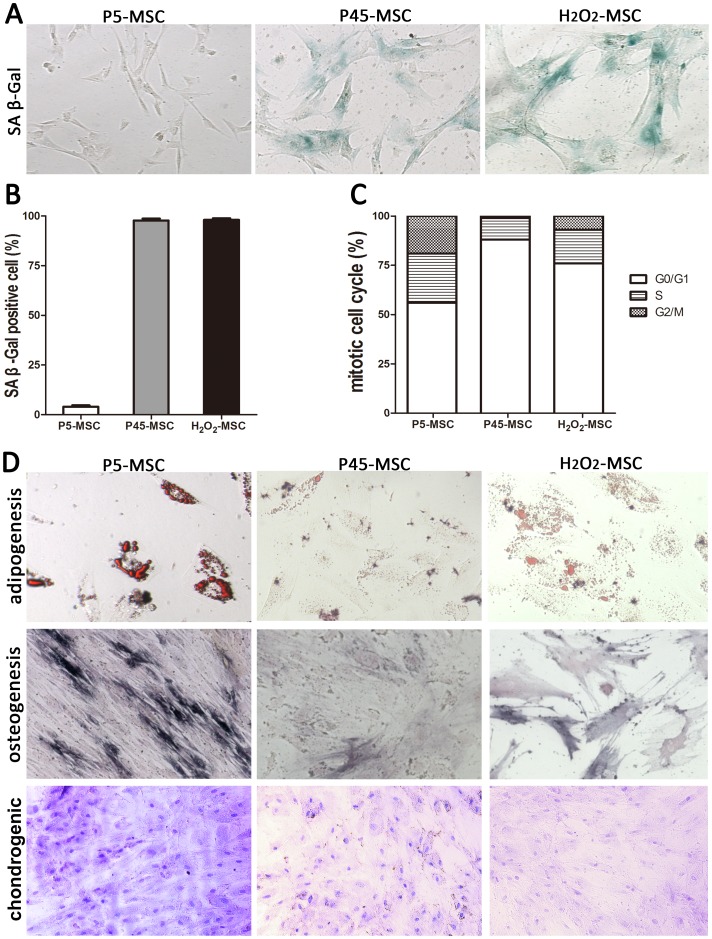
Characterization of s-UCMSCs. (A) Isolated UCMSCs were cultured under normal conditions for several passages until senescent (P45-MSC) or incubated for 2 h in the presence of 200 µM hydrogen peroxide. H_2_O_2_-MSC. The β-galactosidase positive stained cells were quantified as described in (B). (C) Flow-cytometry analysis of the cell cycle distribution of P5-MSC, P45-MSC and H_2_O_2_-MSC. (D) ALP, Oil-Red-O and Toluidine blue staining. Young counterparts (P5-MSC) were used as control.

Isolated human UCMSCs were subcultured serially in platelet lysate supplemented medium. After a certain number of passages (passage 45 in our data; Fig. 1A), MSCs entered a senescent state and senescence-associated features appeared. Cells displayed a characteristic enlarged, flattened morphology, with the accumulation of granular cytoplasmic inclusions (Fig. 1A), irreversible G0/G1 growth arrest (Fig. 1C), and greater than 95% of cells were positive for senescence-associated β-galactosidase staining (Fig. 1B). Similarly, H_2_O_2_ could induce MSCs to senescence. Young MSCs (P5-MSCs) treated with sub-lethal doses of H_2_O_2_ (200 µM) showed characteristic features of senescence regarding morphology and positive β-galactosidase staining (Fig. 1).

The multi-potential of the young and senescent MSCs was analyzed with regard to the adipogenic, osteogenic and chondrogenic differentiation potential. Cells were cultured in specific differentiation media, and 14 days later, the degree of differentiation was assessed by measuring the amount of lipid droplet using Oil-Red-O staining. The result showed that the adipogenic potential sharply declined for senescent MSCs. Similar results were obtained in our study for osteogenic differentiation. After 21 days of osteogenesis, the osteogenic differentiation was evaluated by the staining of cells using a BCIP/NBT kit to examine alkaline phosphatase (ALP) activity. In contrast to young MSCs, senescent MSCs showed a decrease of positive ALP staining. Morover, After induction with chondrogenic medium for 2 weeks, toluidine blue staining revealed less glycosaminoglycans deposition in senescent MSCs (Fig. 1D).

### Senescent UCMSCs promote breast cancer cell migration and proliferation

**Figure 2:**
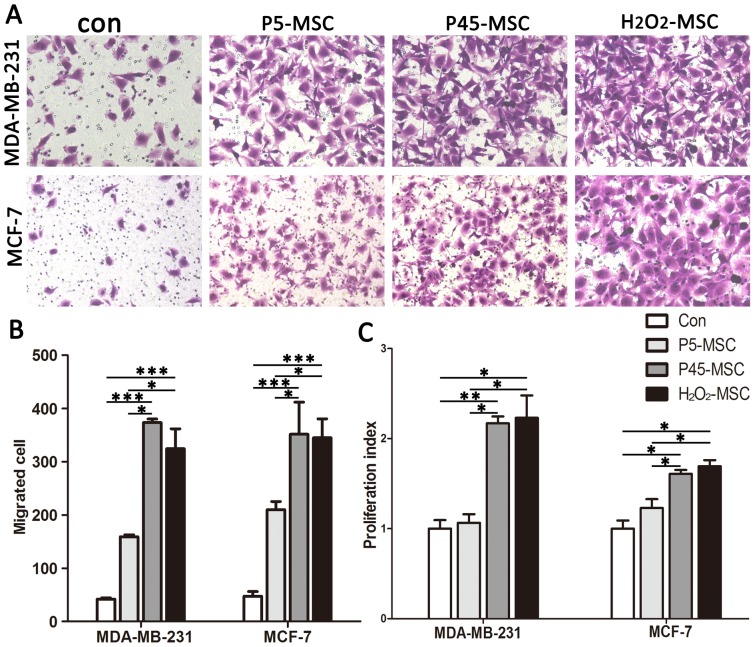
s-UCMSCs promote migration and proliferation of breast cancer cells in vitro. (A) Transwell migration assay. Normal growth medium without cells was used as control. (B) Transmigrated cells were quantified. (C) Proliferation assay. All experiments were repeated at least three times. (*P<0.05; **P<0.01; ***P<0.001).

Senescent MSCs were evaluated in a transwell assay for their ability to induce migration of breast cancer cells with low (MCF-7) or high (MDA-MB-231) invasive capacities. The young MSCs and culture medium alone were utilized as a negative control. MSCs were seeded to a certain density in the lower room of a transwell 36 h before the assay. Breast cancer cells were subsequently placed into the upper room and allowed to move across the porous membrane. As shown in Fig. 2A and B, the invasive capacity of MDA-MB-231 cocultured with senescent MSCs (P45 MSCs) was sharply increased by 8.2 fold in cells compared with culture medium alone (346.7±6.8 vs. 84.4±5.4, respectively). Moreover, H_2_O_2_ induced MSCs were equally proficient at inducing the migration of MDA-MB-231 cells compared with control medium. In contrast, young MSCs (P5 MSCs) only resulted in a modest increase of migrated breast cancer cells (159.2±3.7). A similar result was obtained using MCF-7 cells.

To examine the effect of senescent MSCs on breast cancer cell proliferation, conditioned medium was harvested and used as described before (see in materials and methods). As shown in Fig. 2C, the number of MDA-MB-231 cells in the wells with senescent MSC-CM was significantly higher compared with cells in culture medium only or young MSC-CM. Moreover, enhancement of in vitro growth by senescent MSC-CM was observed in MCF-7 cancer cells.

### IL-6/STAT-3 signal pathway contributes to the senescent MSCs stimulation of breast cancer cells growth and migration

**Figure 3:**
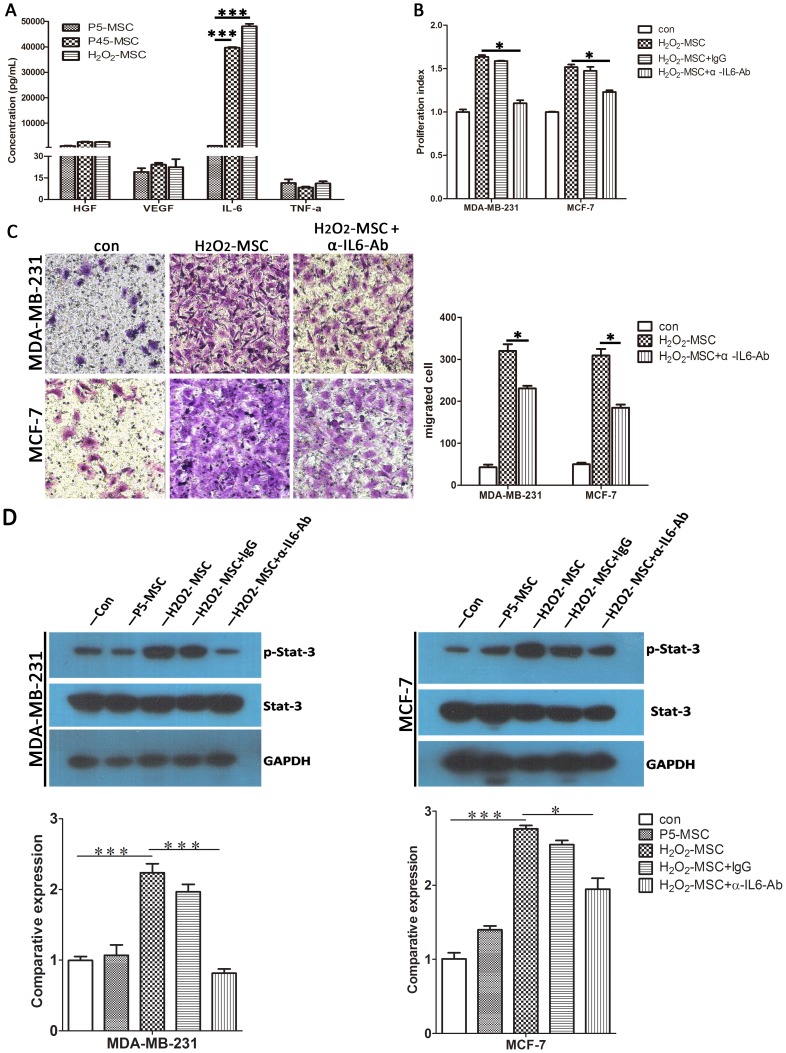
IL-6/STAT-3 signal pathway contributes to the senescent MSCs stimulation of breast cancer cells growth and migration. (A) Protein level of IL-6, HGF, VEGF and TNF-α in the conditioned medium of senescent MSCs (P45-MSC and H_2_O_2_-MSC) and young counterparts (P5-MSC). (B) Proliferation assay. (C) Trans-migration assay of MDA-MB-231 cells and MCF-7 cells using control or IL-6-depleted H_2_O_2_-MSC conditioned medium. (D) Protein level of phosphorylated STAT3 upon treatment with H_2_O_2_-MSC conditioned medium with or without IL-6 specific antibody and Histograms show relative expression level from each group. All experiments were carried out at least three times. (*P<0.05; ***P<0.001).

As there was no direct contact between MSCs and breast cancer cells, we speculated that autocrine or paracrine factor(s) secreted from senescent MSCs was most likely responsible for the enhancement. To examine the molecular mechanism by which senescent MSCs stimulate breast cancer cell proliferation and migration, we assessed four cytokines (IL-6, VEGF, HGF and TNF-α) that are closely related to tumor progression by enzyme linked immunosorbent assay (ELISA). Among them, IL-6 secretion of senescent MSCs was dramatically increased by 40 fold compared with young MSCs (Fig. 3A), strongly suggesting that IL-6 was involved in the ability of senescent MSCs to promote cancer cell growth and migration. As a pleiotropic cytokine, IL-6 is associated with the development and progression of multiple types of cancer. To confirm this hypothesis, the main downstream effector, STAT-3, was activated in both cancer cells when cultured under senescent MSC-CM (Fig. 3D). Furthermore, anti-IL-6 antibodies partially abrogated senescent MSC-CM-induced enhancement of tumor cell proliferation, migration and STAT-3 phosphorylation (Fig. 3B, C and D).

### Senescent MSCs stimulate growth and angiogenesis of breast tumor cells in a xenograft model

**Figure 4:**
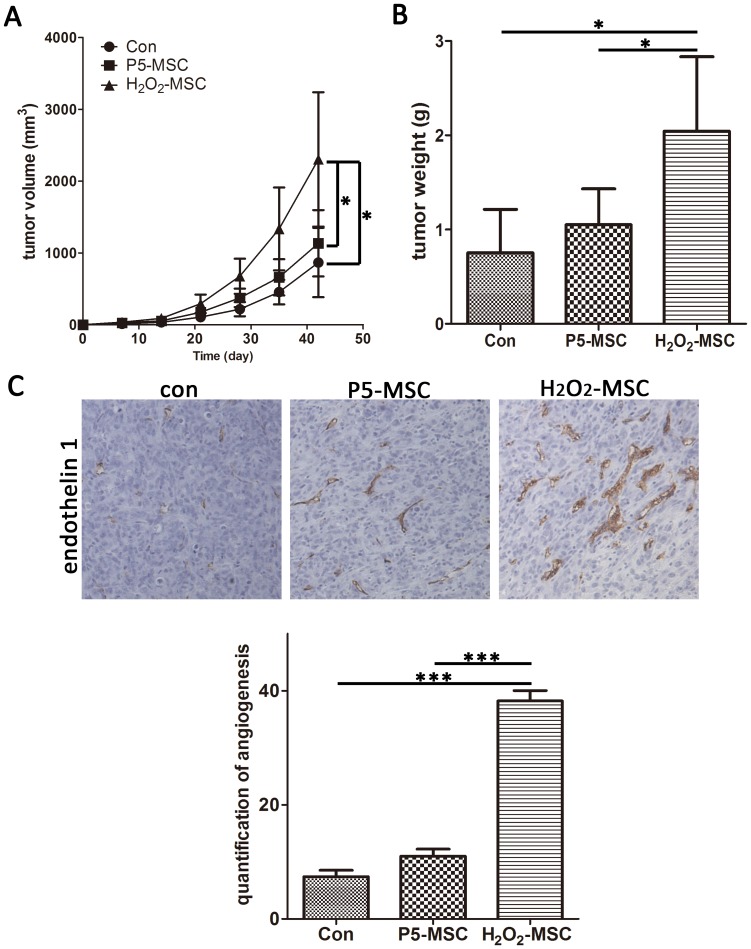
Senescent MSCs stimulate growth and angiogenesis of breast tumor cells in vivo. Tumor volume (A) and final tumor weight (B) were measured as described. (C) Histology and immunohistochemistry for endothelin-1 in tumors derived after a 6-week injection with MDA-MB-231 cells or MDA-MB-231 plus H_2_O_2_-MSC or P5-MSC. (D) Quantification of endothelin-1-positive vessels from each tumor group (*P<0.05; ***P<0.001).

To verify the senescent MSCs stimulative effect on tumor growth in vivo, we injected MDA-MB-231 cells sc in nude mice either alone or mixed with equal numbers of senescent MSCs or young MSCs. In contrast to P45-MSCs, H_2_O_2_-MSCs were easily to be harvested. In additon, it was demonstrated recently that reactive oxygen species (ROS) signaling in the tumor microenvironment induces a form of “accelerated aging”, which leads to stromal inflammation and changes in cancer cell metabolism [20]. So here we used H_2_O_2_-induced MSCs as the senescent cell model. At day 3, it was possible to palpate solid nodules beneath the skin. Interestingly, all the mice (8/8) inject with the senescent MSCs (H_2_O_2_-induced MSCs) developed palpable nodules, whereas only 4/8 and 3/8 of young MSCs (P5-MSC) or control, respectively, could be detected, indicating that senescent MSCs promote tumor initiation. In addition, senescent MSCs not only enhanced tumor formation but also accelerated tumor growth. External caliper measurements showed that MDA231/H_2_O_2_-MSCs xenografts were strikingly larger than MDA231/P5-MSCs and MDA231 xenografts (Fig. 4A). Furthermore, tumor weight of the senescent cell group was significantly higher than the control group (Fig. 4B). We further investigated tumor neovascularization by immunostaining for endothelin-1, an endothelial-specific marker. As shown in Fig. 4B and C, we noted significant augmentation in vascularization when senescent MSCs were co-injected with MDA-231. In addition, both the number and size of endothelin-1-positive vessels were increased by the co-injection of senescent MSCs.

## Discussion

In the present study, we showed that human UCMSCs achieve senescence through replicative exhaustion or a sub-lethal dose of oxidative stress. Our data revealed that senescent MSCs develop a characteristic senescence-associated secretory phenotype (SASP). Senescent MSCs, much more than pre-senescent counterparts, stimulate proliferation and migration of breast cancer cells. Moreover, this cancer-promoting activity was due at least in part to the IL-6/STAT-3 signaling pathway.

With the increased use of MSCs in clinical trials of regenerative medicine and immunomodulatory disorders, the bottleneck for clinical application of MSCs is standardized process of cell preparations and reliable quality control of cell products [21]. Although hMSCs are considered stem cells based on their self-renewing and multi-differentiation capability, they do have a definitive life span and enter into senescence after a certain number of cell divisions. Various critical molecular switches of cellular senescence, such as progressive shortening of telomeres and accumulation of the cyclin-dependent kinase inhibitor, p16INK4a, have been demonstrated for MSCs after repeated passage [22]. Besides the intrinsic factors, the expansion medium and culturing oxygen concentration might also have an enormous impact on the extent of MSCs expansion in vitro [23], [24]. Therefore, the time course of senescence for MSCs varies between different laboratories. Intriguingly, our data revealed that platelet growth factor-enriched human plasma represents an efficient alternative to FCS. MSCs expanded in PL-supplemented medium were prone to maintain stem cell characteristics and retard the aging process, as the typical senescent phenomenon did not arise until 40 passages (Fig. 1A).

It has been reported that MSCs appear to be particularly sensitive to elevated ROS levels [25]. ROS, such as oxygen ions, hydroxyl radicals and peroxide, are highly reactive and cause DNA and cell damage. Similarly to fibroblasts, sub-lethal doses of oxidative stress declined proliferation rates and induced senescent morphological features as well as senescence-associated β-galactosidase positivity (Fig. 1A and B). However, the expression level of surface markers for senescent MSCs remained similar to normal MSCs (data not shown), whilst the osteogenic and adipogenic differentiation capacity dropped (Fig. 1D).

MSCs play a pivotal role in breast cancer progression and metastasis. When recruited into breast tumor stroma, the ‘hijacked’ MSCs tended to be ‘educated’ by tumor cells and form a major component of the tumor microenvironment and regulate cancer stem cell behavior [14]. Bone marrow derived MSCs have been proven to be an important source and contribute 20% to cancer associated fibroblasts (CAFs) [26]. Furthermore, when exposed to breast tumor conditioned medium, MSCs assume a CAF-like phenotype and gained functional properties of CAFs [27]. Additionally, MSCs secrete a variety of cytokines and growth factors that are known to influence tumor growth, metastasis and angiogenesis. It has been reported that co-injection of MSCs with weakly metastatic human breast carcinoma cells greatly increase their metastatic potency. Furthermore, the enhanced metastatic ability was dependent on paracrine factor CCL5 [15]. Moreover, tumor-resident MSCs directly impair the function of a variety of immune cells, including B and T lymphocytes, and natural killer cells [28]. However, unlike bone marrow derived MSCs, human UC-MSCs exerts strong inhibitory effect on several types of tumor cells, possibly through downregulation of PI3K/AKT signaling and activation of p38 MAPK pathway [29]–[31]. In the present study, we demonstrated that the physiological status of UC-MSCs exert crucial influence on its interaction with breast cancer cells. We found that only senescent MSCs showed marked growth and metastasis promotion. In contrast, normal MSCs were only found to create a moderate supportive effect.

Normal cellular senescence prevents neoplastic transformation of damaged cells by imposing an essentially irreversible growth arrest. Paradoxically, senescent cells also promote neoplastic transformation of neighboring epithelial cells [32]. Upon senescence, such cells develop a complex SASP. The altered expression of inflammatory factors, growth cytokines and matrix metalloproteinases could disrupt tissue architecture and function, and then, create a microenvironment that facilitates the growth and progression of the mutant epithelial cells [33]. Interestingly, this secretory phenotype resembles that of CAFs, although senescent fibroblasts and CAFs differ in morphology, growth potential and other traits.

Of the myriad SASP factors associated with inflammation and malignancy, our data revealed that IL-6 is apparently specific for senescent MSCs to promote tumor progression. The IL-6 secretion level of senescent MSCs was found to increase 40 fold, whereas three other cytokines, HGF, VEGF and TNF-α, all of which are closely related to tumor progression, did not show an increase (Fig. 3A). Furthermore, conditioned medium from senescent MSCs could activate STAT3, the main downstream transcription factor of IL-6, in breast cancer cells, and the activation could be blocked by an IL-6-neutralizing antibody. Interestingly, previous studies also demonstrated that IL-6 contributes to many age-related pathologies, including late life cancers [34], and IL-6 secreted from MSCs has been regarded as a crucial cytokine for breast cancer cell metastasis [35].

In summary, our data suggests that cellular senescence and aging of MSCs is similar to the “The Sword of Damocles” and may jeopardize the use of MSCs for therapeutic purposes. Assessment of the overall senescence status of hMSCs should be implemented before clinical application.
